# Validation of Structures in the Protein Data Bank

**DOI:** 10.1016/j.str.2017.10.009

**Published:** 2017-12-05

**Authors:** Swanand Gore, Eduardo Sanz García, Pieter M.S. Hendrickx, Aleksandras Gutmanas, John D. Westbrook, Huanwang Yang, Zukang Feng, Kumaran Baskaran, John M. Berrisford, Brian P. Hudson, Yasuyo Ikegawa, Naohiro Kobayashi, Catherine L. Lawson, Steve Mading, Lora Mak, Abhik Mukhopadhyay, Thomas J. Oldfield, Ardan Patwardhan, Ezra Peisach, Gaurav Sahni, Monica R. Sekharan, Sanchayita Sen, Chenghua Shao, Oliver S. Smart, Eldon L. Ulrich, Reiko Yamashita, Martha Quesada, Jasmine Y. Young, Haruki Nakamura, John L. Markley, Helen M. Berman, Stephen K. Burley, Sameer Velankar, Gerard J. Kleywegt

**Affiliations:** 1 Protein Data Bank in Europe (PDBe), European Molecular Biology Laboratory, European Bioinformatics Institute (EMBL-EBI), Wellcome Genome Campus, Hinxton, Cambridge CB10 1SD, UK; 2 RCSB Protein Data Bank, Center for Integrative Proteomics Research, Rutgers, The State University of New Jersey, Piscataway, NJ 08854, USA; 3 BMRB, BioMagResBank, University of Wisconsin-Madison, Madison, WI 53706, USA; 4 PDBj, Institute for Protein Research, Osaka University, Osaka 565-0871, Japan; 5 RCSB Protein Data Bank, San Diego Supercomputer Center, University of California San Diego, La Jolla, CA 92093, USA; 6 Skaggs School of Pharmacy and Pharmaceutical Sciences, University of California San Diego, La Jolla, CA 92093, USA; 7 Institute for Quantitative Biomedicine, Rutgers, The State University of New Jersey, Piscataway, NJ 08854, USA; 8 Rutgers Cancer Institute of New Jersey, Rutgers, The State University of New Jersey, New Brunswick, NJ 08903, USA

**Keywords:** PDB, wwPDB, validation, structure data quality, data archiving, 3D macromolecular structure, structural biology, data deposition, biocuration

## Abstract

The Worldwide PDB recently launched a deposition, biocuration, and validation tool: OneDep. At various stages of OneDep data processing, validation reports for three-dimensional structures of biological macromolecules are produced. These reports are based on recommendations of expert task forces representing crystallography, nuclear magnetic resonance, and cryoelectron microscopy communities. The reports provide useful metrics with which depositors can evaluate the quality of the experimental data, the structural model, and the fit between them. The validation module is also available as a stand-alone web server and as a programmatically accessible web service. A growing number of journals require the official wwPDB validation reports (produced at biocuration) to accompany manuscripts describing macromolecular structures. Upon public release of the structure, the validation report becomes part of the public PDB archive. Geometric quality scores for proteins in the PDB archive have improved over the past decade.

## Highlights

• Validation reports are available for X-ray, NMR, and EM structures in the PDB
• Preliminary reports obtained at deposition, stand-alone servers, and programmatically
• Official reports from biocuration should be submitted with manuscripts
• Quality metrics for protein structures have improved over the past decade

## Graphical Abstract

**Figure:**
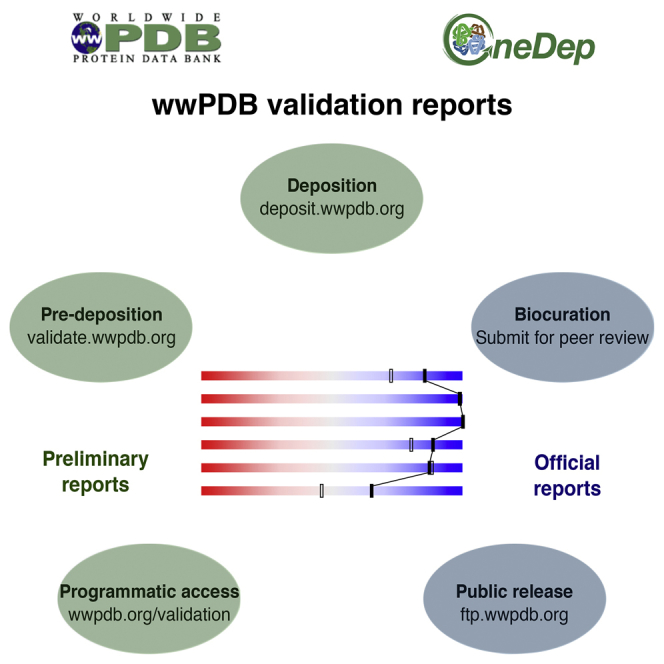

## Teaser

Gore et al. describe the community-recommended validation reports, produced by wwPDB at deposition and biocuration of PDB submissions, and integrated into the archive of publicly released PDB entries. The authors also show that the quality of protein structures has improved over the last decade.

## Introduction

The Worldwide Protein Data Bank (wwPDB; https://wwpdb.org [Berman et al., 2003]) is the international consortium that maintains the Protein Data Bank—the single global archive of three-dimensional (3D) structural models of biological macromolecules and their complexes as determined by X-ray crystallography (89% of holdings as of 15 March 2017), nuclear magnetic resonance (NMR) spectroscopy (9%), three-dimensional cryoelectron microscopy (3DEM, 1%), and other techniques (<1%). wwPDB consortium members include the Research Collaboratory for Structural Bioinformatics (RCSB PDB; Berman et al., 2000), Protein Data Bank in Europe (PDBe; Velankar et al., 2016), Protein Data Bank Japan (PDBj; Kinjo et al., 2017), and Biological Magnetic Resonance DataBank (BMRB; Ulrich et al., 2008). In an effort to improve efficiency and share the structure deposition workload, the four wwPDB partners recently launched OneDep, a unified system for deposition, biocuration, and validation of macromolecular structure data (Young et al., 2017). The biocuration of PDB entries primarily involves verification, consistency checking, and standardization of submitted data. Biocurators review and annotate polymer sequence information, chemical description of ligands and modified polymer residues, and composition of biological assemblies.

In structural biology it has become critically important to supply experimental data along with atomic coordinates to allow validation of the structural model and to support inferences therefrom. Clearly, raw experimental data, before application of any transformations which may lead to loss of information, and devoid of interpretation, would lend the ultimate support of the final model and allow an independent verification of the results, leading to novel validation tools. Efforts to archive such raw data are under way through established archives for X-ray diffraction images (Meyer et al., 2016, Grabowski et al., 2016), X-ray free electron laser images (Maia, 2012), NMR free induction decay (Ulrich et al., 2008), and 3DEM images (Iudin et al., 2016). The wwPDB currently enforces archiving of reduced representations of experimental data (structure-factor amplitudes or intensities for crystallography, chemical shifts and various types of restraints for NMR, and reconstructed volume maps and tomograms for 3DEM), while encouraging deposition of raw experimental data into these method-specific resources. Efforts by the PDBx/mmCIF Working Group to improve and extend the capture of processed diffraction data to include unmerged intensities and details of crystal samples and raw images contributing to integrated intensities are ongoing. Mandatory archiving of structure factors and NMR restraints began in 2008 (Dutta et al., 2009), followed by NMR-assigned chemical shifts in 2010, and 3DEM volume maps in 2016. The availability of experimental data not only enhances the integrity of the PDB archive but also allows systematic validation of atomic structures, and ultimately leads to better validation tools and improved quality of the archived data. Validation tools developed by the community and implemented within the OneDep system help to identify possible issues with experimental data, atomic model, or both, and thus allow depositors the opportunity to review and correct any errors prior to concluding a PDB deposition. In addition, unresolved issues may be uncovered by wwPDB biocurators or by manuscript reviewers, who are provided with access to the official wwPDB validation report. One of the more time-consuming tasks faced at present by wwPDB biocurators is the reprocessing of entries, as occasioned by depositors submitting revised atomic models to address issues uncovered during biocuration or manuscript peer review. The wwPDB stand-alone validation server (https://validate.wwpdb.org) was developed with the express purpose of enabling depositors to identify problems and resolve them in advance of submission.

To incorporate state-of-the-art validation tools into the wwPDB biocuration pipeline, and to provide useful validation metrics to depositors and other PDB users, the wwPDB convened Validation Task Forces (VTFs) for crystallography (Read et al., 2011) and NMR (Montelione et al., 2013), and together with the EMDataBank project partners (Lawson et al., 2016) convened a corresponding VTF for 3DEM (Henderson et al., 2012). A validation software pipeline informed by the recommendations of the three VTFs has been integrated into both the OneDep system (https://deposit.wwpdb.org) and the stand-alone wwPDB validation server (https://validate.wwpdb.org).

All three VTFs recommended that structures deposited to the PDB be validated against three broad categories of criteria, each of which is discussed in more detail in subsequent sections. The first category involves knowledge-based validation of the atomic model, without regard to the associated experimental data. Examples include the number of residues that are outliers in the Ramachandran plot (Ramachandran et al., 1963) and the number of too-close contacts (clashes) between non-bonded atoms. For each of these criteria, the report provides both raw (number of outliers) and normalized (percentage of outliers) scores. To the extent possible, structural models from all experimental methods are evaluated with the same criteria in this category. The second category involves analysis of experimental data (independent of the derived atomic coordinates). Criteria in this category are specific to the experimental technique and sometimes to its “submethods”; they include metrics such as Wilson B value (Wilson, 1948) or estimated twinning fraction (Padilla and Yeates, 2003) in crystallography and completeness of chemical-shift assignments in NMR. The third category involves analysis of the fit between the atomic coordinates and the underlying experimental data. Criteria for crystallography include metrics such as *R* and *R*_free_ (Brünger, 1992) and real-space-fit outlier residues. Criteria for NMR and 3DEM models are still under development, and the validation pipeline will be augmented with these when they become available. Some metrics are analyzed across the entire archive so that percentile scores can be derived.

It is very important to note that issues highlighted by a validation metric do not necessarily imply errors in the model. Instead they may point to genuine, albeit unusual, features of the structure, which may be of biological interest: e.g., Val50 in the structure of the protein annexin (PDB: 2HYV; Shao et al., 2006) is involved in Ca^2+^ ion coordination and is consistently flagged as a Ramachandran outlier. Such unusual features should, however, be supported by convincing experimental evidence. The wwPDB is working toward providing depositors with a mechanism for adding explanatory comments to the official wwPDB validation reports.

## Results

### Validation Report Content

Official wwPDB validation reports provide both overall quality scores for a PDB submission and detailed lists of specific issues. Above-average global scores can sometimes mask local issues; hence it is important to review the entire report, especially during structure refinement.

The reports are provided as human-readable PDF files and as machine-readable XML files, and are made available with the public release of the corresponding PDB entry. The machine-readable files contain all of the detailed validation information and statistics. For example, the validation XML file specifies for each protein residue any outlying bond length or bond angle, the residue's rotameric state, its region in a Ramachandran plot (Ramachandran et al., 1963, Chen et al., 2010), any atoms involved in too-close contacts, and (for X-ray structures) the fit to electron density. These XML files can be read and interpreted by popular visualization software packages, such as Coot (Emsley et al., 2010), to display validation information for any publicly available PDB entry.

Herein, we describe the format and content of the PDF files, which are the more commonly accessed validation report files. A full description of the report content is available at https://wwpdb.org/validation/validation-reports. The PDF validation reports are available in two formats: a summary, in which a maximum of five outliers are presented for each metric, and a complete report, in which all outliers are enumerated.

#### Sections of the wwPDB Validation Report

**Figure 1:**
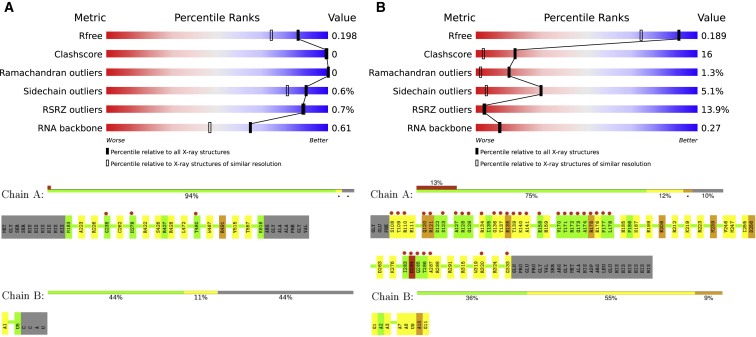
Summary Quality Metrics in the wwPDB Validation Reports Sliders (top) and residue plots (bottom). (A) relatively good structure; (B) relatively poor structure. The solid sliders report on how a given structure ranks relative to all structures in the PDB. The open sliders report on the comparison with structures derived in a similar fashion (X-ray crystallographic structures are compared with other X-ray structures solved at a similar resolution, while NMR and EM structures are ranked relative to other NMR and EM structures in the PDB, respectively). Residue sequence plots flag residues that have unusual geometry features (i.e., bond length, bond angle, Ramachandran, RNA suiteness, or other torsion-angle outliers). Residues are color coded as follows: green, no geometric outliers; yellow, 1 type of outliers; orange 2 types of outliers; red, 3 or more types of outliers; gray, atomic coordinates not available; cyan, atomic coordinates are ill-defined by the NMR ensemble. For X-ray crystal structures, a red dot above a residue indicates a poor fit to electron density (*RSRZ* > 2).

The PDF reports are organized as follows. The title page displays the wwPDB logo (and also the EMDataBank logo for EM entries), specifies the type of the report (whether it is preliminary, confidential, or produced for a publicly available PDB entry), shows basic administrative information about the uploaded data or the PDB entry, lists the software packages and versions that were used to produce the report, and provides a URL to access help text at https://wwpdb.org. The executive summary (“Overall quality at a glance”) shows key information about the entry, such as the experimental technique employed to determine the structure, a proxy measure of information content of the analyzed data (resolution for crystal and 3DEM structures and completeness of resonance assignments for NMR), and a number of percentile scores (“sliders”), comparing the validated structure to the entire PDB archive (Figure 1). Table 1 lists key criteria reported in this section, covering knowledge-based geometric validation scores. For crystal structures, the fit to experimental data is summarized by an overall measure (*R*_free_ factor) and by the fraction of residues that locally do not fit the electron density well (normalized real-space *R* value, *RSRZ*) (Kleywegt et al., 2004). These criteria were selected because they are not typically optimized directly during structure refinement (unlike, e.g., the conventional *R* value and bond lengths and bond angles) (Kleywegt and Jones, 1995). Ideally, a high-quality structure will score well across the board. Good values for only one of the metrics (e.g., a perfect fit to electron density) with poor scores for others (e.g., many Ramachandran outliers) could be a sign of a biased model building/refinement protocol (e.g., overfitting to experimental data). For each metric, two percentile ranks are calculated: an absolute rank with respect to the entire PDB archive and a relative rank. For crystallographic structures, the relative rank is calculated with respect to structures of similar resolution (at least 1,000 structures), while structures derived from NMR or 3DEM are compared against all other NMR or 3DEM structures, respectively. Absolute percentile scores are useful to general users of the PDB to evaluate whether a given PDB entry is suitable for their purposes, while the relative percentiles provide depositors, editors, reviewers, and expert users with a means to assess structure quality relative to other structures derived in a similar manner.

**Table 1:** Key Validation Metrics Reported in the wwPDB Structure Validation Reports and Used for Percentile Rank Calculation

Metric	Details	Software Package and References
*R*_free_	cross-validation of goodness of fit between the model and the experimental diffraction data not used for refinement. Applicable to crystallographic structures	DCC (Yang et al., 2016)
Clashscore	number of too-close contacts in an entry normalized per 1,000 atoms	MolProbity (Chen et al., 2010)
Ramachandran outliers	fraction of polypeptide residues deemed to have very unusual backbone conformation (<0.5% of those observed in a high-quality reference set)	MolProbity (Chen et al., 2010), Maxit (Z.F., https://sw-tools.rcsb.org/apps/MAXIT)
Side-chain outliers	fraction of polypeptide residues in non-rotameric side-chain conformations (<0.5% of those observed in a high-quality reference set)	MolProbity (Chen et al., 2010)
*RSRZ* outliers	fraction of polypeptide and/or polynucleotide residues that do not fit the electron density well when compared with other instances of the same residues in structures at similar resolution. Applicable to crystallographic structures	EDS (Kleywegt et al., 2004)
RNA backbone	average score over all RNA nucleotides in the entry indicating the quality of the observed RNA backbone conformation	MolProbity (Chen et al., 2010)

The percentile ranks are followed by a graphical summary of chain quality (Figure 1). Each standard polypeptide and polynucleotide residue is checked against ideal bond and angle geometry, torsion-angle statistics, and contact distances. Residues are then color coded based on the results: green if no issues are detected, yellow if there are outliers for one criterion (e.g., unusual bond lengths), orange if there are outliers for two criteria (e.g., unusual bond lengths and too-close contacts), and red for three or more criteria with outliers reported. A horizontal stack bar plot presents the fraction of residues with each color code for each polypeptide or polynucleotide chain. The fraction of residues present in the experimental sample but not included in the refined atomic model is represented by a gray segment, and the fraction of residues “ill-defined” by the NMR ensemble (see below) is represented by a cyan segment. For X-ray crystal structures, an upper red bar indicates the fraction of residues with a poor fit to the electron density. This is followed by a table listing ligand molecules that show unusual geometry, chirality, and/or fit to the electron density.

The section on overall quality is followed by one on entry composition, which describes each unique molecule present in the entry. For NMR entries, a separate section on ensemble composition is also included. As most NMR structures are deposited as ensembles of conformers, this section reports on what parts of the entry are deemed to be well-defined or ill-defined (Kirchner and Güntert, 2011) and also identifies a medoid representative conformer from the ensemble, i.e., the conformer most similar to all the others (Montelione et al., 2013).

The section on residue quality highlights residues that exhibit at least one kind of issue, i.e., color coded yellow, orange, or red, as described above (Figure 1). While unusual features (e.g., a residue falling into a disallowed region of the Ramachandran plot) are not unexpected even in high-resolution structures, typically occurring with a frequency of 0.5% (Chen et al., 2010), they nevertheless should be inspected, and the sequence plots are intended to help users more easily find residues with validation issues.

The section that presents an overview of the experimental data is specific to each experimental technique. For X-ray crystal structures, the structure factors are analyzed using the Phenix tool Xtriage (Adams et al., 2010) to identify outliers, assess whether the crystalline sample was twinned, and analyze the level of anisotropy in the data. The *R* and *R*_free_ values are presented as provided by the depositor and as recalculated by the wwPDB from structure-factor amplitudes and the model. The *R*_free_ value measures how well the atomic model predicts the structure factors for a small subset of the reflections (typically 5%–10%) that were not included in the refinement protocol (Brünger, 1992). It is a useful validation metric showing whether there are sufficient experimental data and restraints compared with the number of adjustable parameters in the model: *R*_free_ values much higher than *R* could indicate an overfitting to experimental data during refinement. *R* values provided by the depositor are displayed along with *R* values recalculated by the DCC tool (Yang et al., 2016) from the atomic model and structure factors with the same refinement program as was used to refine the atomic model. Good agreement between the depositor *R* values and those recalculated serves to check whether the data have been uploaded and interpreted correctly within the OneDep system.

For NMR structures, the report contains an overview of the structure determination process and the overall completeness of the resonance assignments. For 3DEM structures, if a volume map is available, basic information describing the experimental setup and the map is included.

The section on model validation provides further details for each criterion covering polypeptides, ribonucleic acids, small molecules, and non-standard polymer residues. The bond lengths and bond angles of amino acid and nucleotide residues are checked by MolProbity's Dangle module (Chen et al., 2010) against standard reference dictionaries (Engh and Huber, 2001, Parkinson et al., 1996). Close contacts between non-bonded atoms are analyzed using MolProbity. As MolProbity does not deal with close contacts between symmetry-related molecules in the case of crystallographic experiments, these are checked by the in-house software “MAXIT” (Z.F., https://sw-tools.rcsb.org/apps/MAXIT/index.html). MolProbity also performs protein-backbone and side-chain torsion-angle analysis (Ramachandran plot and rotameric state) and RNA-backbone and ribose-pucker analysis. For X-ray crystal structures of proteins, cases where 180° flips of histidine rings and glutamine or asparagine side chains improve the hydrogen-bonding network without detriment to the electron density fit are also reported. The MAXIT software is also used to identify and report *cis*-peptides and stereochemistry issues, such as chirality errors and polymer linkage artifacts.

The geometry of all non-standard or modified residues of a polymer, small-molecule ligands, and carbohydrate molecules is analyzed with the Mogul software (Bruno et al., 2004). For each bond length, bond angle, dihedral angle and ring pucker, Mogul searches through high-quality, small-molecule crystal structures in the Cambridge Structural Database (CSD) (Groom et al., 2016) to identify similar fragments. Each bond length, angle, and so forth in the compound is compared against the distribution of values found in comparable fragments in the CSD, and outliers are highlighted. Chirality problems are diagnosed by checking against the wwPDB Chemical Component Dictionary definitions (Westbrook et al., 2015).

The fit of the atomic model to experimental data (currently only available for X-ray crystal structures) is analyzed by the procedure developed for the Uppsala Electron Density Server (Kleywegt et al., 2004). Electron density maps are calculated with the REFMAC program (Murshudov et al., 1997) using the atomic model and the structure factors. The fit is assessed between an electron density map calculated directly from the model (DF_calc_ map) and one calculated based on model and experimental data (2mF_obs_-DF_calc_ map). The fit is analyzed on a per-residue basis for proteins and polynucleotides, and reported as the real-space *R* value (*RSR*) (Jones et al., 1991). These *RSR* values are normalized by residue type and resolution band to yield *RSRZ* (Kleywegt et al., 2004). Residues with *RSRZ* >2 are reported as outliers. At present, this analysis is not possible for non-standard amino acids/nucleotides or ligands, as these compounds are not present in sufficient numbers in the PDB to generate reliable *Z* scores. For these, therefore, only the *RSR* value, real-space correlation coefficient, and the so-called Local Ligand Density Fit score (*LLDF*) are reported. *LLDF* for a ligand or non-standard residue is calculated as follows: all standard amino acid or nucleotide residues within 5.0 Å distance of any atom of the ligand or non-standard residue are identified by the CCP4 NCONT program, taking crystallographic symmetry into account (Winn et al., 2011). The mean and SD of the *RSR* values for these neighboring residues are then calculated, and these are used with the *RSR* value of the ligand or the non-standard residue itself to provide a local, internal *Z* score. If fewer than two neighboring residues are within 5.0 Å of the entity, then *LLDF* cannot be calculated (this occurs for ∼20% of ligands in PDB entries released before 31 December 2016). *LLDF* values greater than 2 are highlighted in the reports (this occurs for 34% of ligands in PDB entries released before 31 December 2016 for which an *LLDF* value could be calculated) (O.S.S. et al., unpublished data). The wwPDB partners and the crystallography community are evaluating this and other metrics to reliably assess the fit to electron density for bound ligands, following the recommendations of the wwPDB/CCDC/D3R Ligand Validation Workshop (Adams et al., 2016).

For NMR structures, the report contains a section on validation of assigned chemical shifts. Each structure can potentially be linked to more than one list of chemical shifts (e.g., from samples with different experimental conditions or isotope labeling pattern). Therefore, each chemical-shift list is treated independently. For each list, a table summarizing any parsing and mapping issues between the chemical shifts and the model coordinates helps depositors detect and correct data entry errors. For entries containing proteins, the PANAV package (Wang et al., 2010) is invoked to suggest corrections to chemical-shift referencing. Completeness of resonance assignments per chemical-shift list is calculated for each type of nucleus and location (e.g., backbone, aliphatic or aromatic side chain). Unusual chemical-shift assignments are identified according to the statistics compiled by BMRB (Ulrich et al., 2008). Severe chemical-shift outliers (e.g., >30 SDs from the average value) are frequently the result of spectral “aliasing,” and these need to be corrected to achieve valid data deposition. Finally, for entries containing polypeptides, the amino acid sequence and chemical shift information is used by the RCI software (Berjanskii and Wishart, 2005) to calculate a random coil index (*RCI*) for each residue, which estimates how likely the residue is to be disordered. In a bar-graph representation of *RCI* for each polypeptide chain, each residue considered to be ill-defined from the analysis of the NMR ensemble of conformers (see above; Kirchner and Güntert, 2011) is colored cyan; this result from analysis of coordinates alone can then be compared with experimental evidence for potential disorder from the *RCI*.

### Implementation and Delivery

#### Usage of the wwPDB OneDep Validation Pipeline

**Figure 2:**
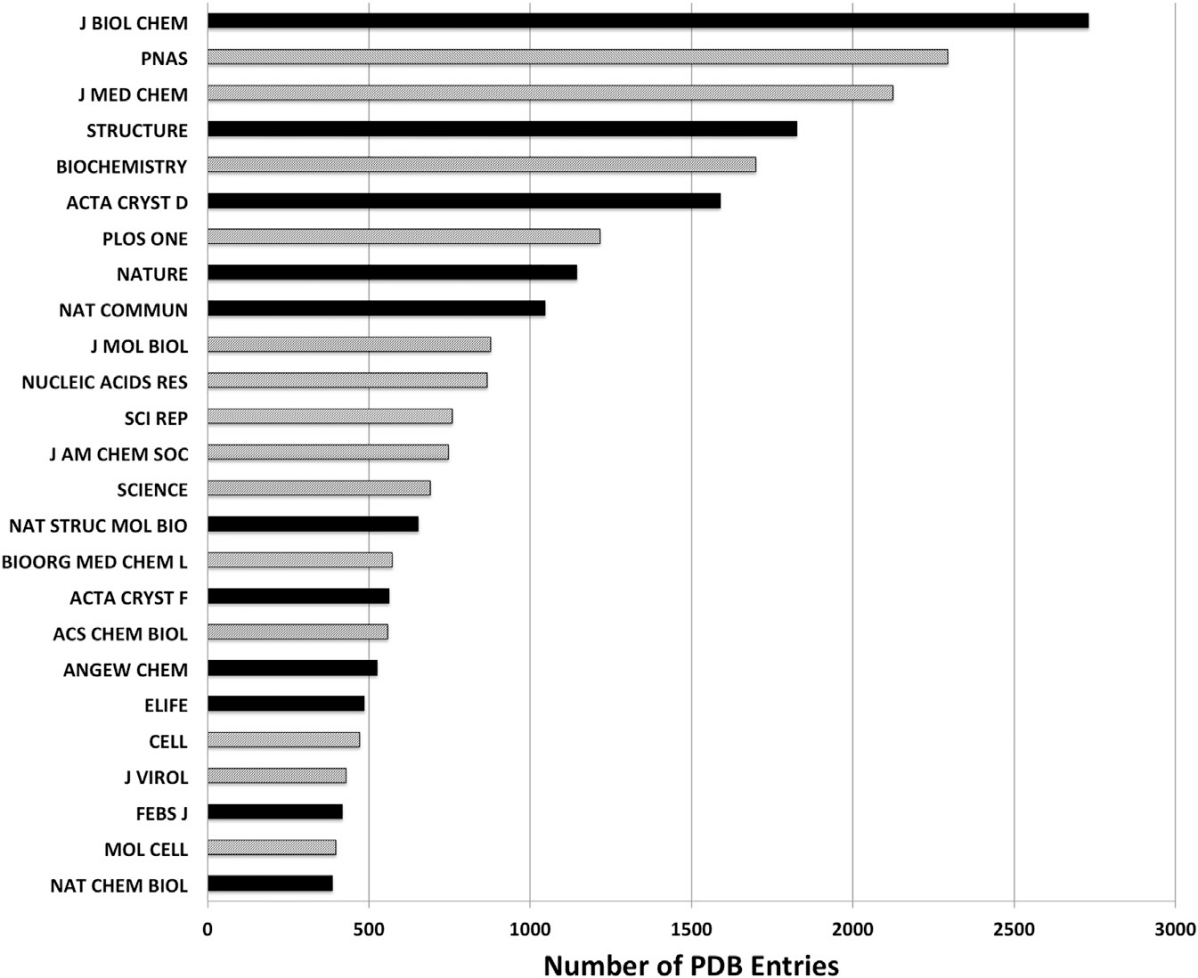
List of 25 Journals, which Publish Most Papers Describing PDB Structures, Ranked According to Their Citation in the PDB from 2012 to 2016 Journals that require wwPDB validation reports for manuscript review are shown in black, while the ones that do not yet require the reports are shown in gray. Note that obsoleted entries are only included when calculating these statistics if they were superseded by a different PDB entry. Obsoleted (retracted) entries were excluded.

The OneDep validation module is used at various points during PDB data deposition and biocuration (Table 2). When data deposition is concluded, a preliminary validation report is supplied to the depositor, who must review and accept this report before the uploaded data can be submitted for biocuration. Depositors are strongly encouraged to review all issues enumerated in the preliminary validation report and to address them before continuing to the submission step. Data re-upload is possible at this stage in the process. Once the depositor accepts the preliminary validation report, uploaded data are submitted for biocuration, which serves to resolve data integrity and representation issues prior to the final validation step, which results in the official wwPDB validation report for the uploaded entry. These official wwPDB validation reports are watermarked as confidential and contain information describing the entry, including title and PDB accession code, plus a much richer analysis of small molecules and non-standard polymer residues than is possible at the preliminary stage. A growing number of journals (Figure 2) require that manuscripts describing biomacromolecular structures be accompanied by the official wwPDB validation report. At the time of public release of the entry, the official wwPDB validation report is updated to reflect any revisions to the entry or to the validation pipeline. Released official wwPDB validation reports are made publicly available via the wwPDB FTP area and the wwPDB partner websites. Population statistics for the entire archive are recalculated annually and the reports for all entries are then updated accordingly (see below).

**Table 2:** Modes of Validation Pipeline Invocation

Mode of Execution	Distinct Features of the Report	Access
Web-service API	preliminary, as input files may not have final nomenclature, optionally accepts experimental data. Watermarked “Preliminary”	installation instructions at https://wwpdb.org/validation/onedep-validation-web-service-interface
Stand-alone web server	preliminary, optionally accepts experimental data. Watermarked “Preliminary”	https://validate.wwpdb.org
Deposition interface	preliminary, contains deposition session identifier, requires experimental data for X-ray crystal, NMR, and EM structures. Watermarked “Preliminary” depositor must review and accept prior to submission	https://deposit.wwpdb.org
Biocuration	complete, as the input files have been updated to conform to PDB standards and nomenclature, confidential, recommended by wwPDB to accompany manuscript submissions, contains PDB entry code and title. Watermarked “Confidential”	only accessible by wwPDB biocurators
Public release	complete, includes PDB entry code, title, and authors. Not watermarked	ftp://ftp.wwpdb.org and wwPDB partner websites

The same validation module is available from the wwPDB stand-alone validation web server and from an application programming interface (API) designed for use by structure determination, refinement, and visualization software (Table 2). The primary function of the stand-alone validation web servers and the API is to allow checking of the atomic model and experimental data during structure determination and refinement. At the time of writing, these two access modes combined generate on average ∼600 invocations of the wwPDB validation pipeline per week. We expect this number to increase as awareness builds in the community. At present, the wwPDB stand-alone validation web servers/API generate only preliminary wwPDB validation reports, which are not appropriate for submission with scientific manuscripts.

#### Implementation Details

The wwPDB validation pipeline orchestrates execution of each community-recommended validation tool (Table 3), extracts key metrics produced by these tools, and packages this information in both summary reports and detailed XML data files (Gore et al., 2012). The pipeline is implemented as a set of modules, each responsible for preparing the inputs in required formats and parsing the outputs of a particular validation tool.

**Table 3:** Component Software Packages Included in the 2017 Version of the Validation Pipeline

Software Package	Which Section and Metric of the Report the Package Is Used for	Reference
MolProbity	model geometry: bond lengths and bond angles of standard protein residues and nucleotides, too-close contacts, Ramachandran outliers, rotamer outliers, RNA suiteness	Chen et al., 2010
MAXIT	model geometry: symmetry-related too-close contacts, stereochemistry issues, identification of *cis*-peptides	Maxit (Z.F., https://sw-tools.rcsb.org/apps/MAXIT/index.html)
Mogul	model geometry: bond-length and bond-angle outliers in small molecules	Bruno et al., 2004
Xtriage (Phenix)	crystallographic data and refinement statistics: signal-to-noise, twinning	Adams et al., 2010
DCC	crystallographic data and refinement statistics: *R*, *R*_free_ fit to crystallographic data: *R*_free_	Yang et al., 2016
EDS	fit to crystallographic data: real-space *R* outliers	Kleywegt et al., 2004
Cyrange	NMR ensemble composition: identification of well-defined protein cores	Kirchner and Güntert, 2011
RCI	NMR chemical shifts: prediction of protein backbone order parameter from chemical shifts	Berjanskii and Wishart, 2005
PANAV	NMR chemical shifts: suggested referencing corrections in chemical shift assignments	Wang et al., 2010

The modules access data and validation tools through a collection of APIs shared by all of the wwPDB OneDep system components. These core APIs provide uniform access to the diverse set of pipeline dependencies, including both locally developed and community-supported tools and libraries. As the pipeline executes each module, it records names and versions of each validation tool together with the completion status for the tool. Pipeline results are recorded in data files and summarized in formatted reports. The data file organization is documented in the XSD format schema files (https://wwpdb.org/validation/schema/wwpdb_validation_v002.xsd). Summary reports are composed using TeX formatting instructions and rendered in PDF format for delivery.

Access to the wwPDB validation pipeline is provided in three ways: as an anonymous pre-deposition web user interface, as an integral part of the wwPDB deposition and biocuration platform, and as a web-service API. The web user interface implementation makes use of the OneDep software framework (Young et al., 2017), which selects only the subset of the deposition user interface features required to support the validation service. The anonymous wwPDB stand-alone and OneDep deposition validation services both manage computationally intensive workloads using the OneDep internal workflow system (Young et al., 2017). While both services share the same OneDep software stack, these services are independently deployed and hosted on separate compute clusters. Compute resources can be scaled according to demand.

The web-service API is supported by both a client-side Python implementation and a Unix command-line interface (CLI). Execution of the wwPDB validation pipeline using the API involves multiple steps performed in the context of a validation session. Within a session, the API provides methods to upload data files, queue validation pipeline requests, check completion status, and recover result files. The API steps are summarized in Table 4. The Python client API, bundled by standard Python package management tools (PIP), is available from the Python Package Index server (https://pypi.python.org/pypi/onedep_api/0.15). Installation and user documentation for the Python API and CLI are provided at https://wwpdb.org/validation/onedep-validation-web-service-interface.

**Table 4:** Step-by-Step Flow of Validation Pipeline Web-Service API

Step	Description
Start a new validation session	returns a new unique code to reference subsequent API steps
Upload data files	coordinate models and supporting experimental data files (e.g., X-ray structure-factor amplitudes, NMR chemical shifts)
Submit pipeline execution request	queue the session for execution
Check completion status	return a completion status for the current session (e.g., queued, running, successfully completed, or failed completion)
Session file inventory	return a list of data and result files within the current session
Download output files	recover a session result file

Future resource requirements of the web-service API are anticipated to be significantly greater than those of the web user interfaces. As a result, a different workflow system has been developed to support the web-service deployment. This system uses a message broker to route requests from the web-service API to a distributed collection of task queues. Queued validation task requests are handled by a set of back-end services. The volume of back-end services can be adjusted quickly in response to changes in workload. Our current implementation uses the RabbitMQ (https://www.rabbitmq.com/) message broker and the supporting AMQP (https://www.amqp.org/) Python client library.

#### Annual Recalculation of Percentile Statistics

As the component validation tools and underlying reference datasets of high-quality structures are updated, both raw and normalized scores calculated by the wwPDB validation pipeline are likely to change over time. Moreover, as the PDB continues to grow (11,614 new depositions were received in 2016), percentile ranks of structures also change. To account for such changes, wwPDB validation reports are regenerated annually for the entire public archive, with recalculated statistics underlying the percentile ranks based on the state of the PDB archive on December 31 of the preceding calendar year. Following internal review, the updated reports replace the older versions in the public wwPDB FTP areas. The most recent update took place on March 15, 2017. Older reports continue to be accessible via yearly snapshots of the wwPDB FTP area.

For most entries, changes in the percentile ranks are modest year-on-year. However, with improved tools for structure determination and more awareness of the importance of validation, it is hoped that erroneous features will become increasingly rare in newly deposited structures. As a result, the percentile ranks for older structures are expected to slowly decline, reflecting an increase in overall quality of structures in the PDB archive.

### Journal Interactions

Official wwPDB validation reports provide an assessment of structure quality using widely accepted and community-recommended standards and criteria. To help deliver the best possible quality in the PDB archive, the wwPDB partners strongly encourage journal editors and referees to request these reports from authors as part of the manuscript submission and review process. To achieve this goal, wwPDB partners have formally approached the journals responsible for publishing most structures to request them to implement mandatory submission of official wwPDB validation reports together with manuscripts describing the structures. (n.b.: The official version of the validation report is clearly identified by the watermark [Table 2] and the cover page, which indicates that the report is recommended for journal editors and reviewers. The reports are date-stamped and display the wwPDB logo.) Figure 2 lists the 25 journals that published the majority of PDB structures between 2012 and 2016.

At the time of writing, submission of official wwPDB validation reports is required by *Structure* (http://crosstalk.cell.com/blog/show-us-your-pdb-validation-reports), the *Nature* Publishing Group (Editorial, 2016), *eLife*, the *Journal of Biological Chemistry*, all *International Union of Crystallography* (*IUCr*) journals, *FEBS Journal*, the *Journal of Immunology*, and *Angewandte Chemie International Edition in English* as part of their manuscript submission process. Submission of official wwPDB validation reports is further recommended by *Cell*, *Molecular Cell*, and *Cell Chemical Biology*. The interaction between wwPDB and journals is an ongoing effort. More journals have expressed interest recently, and we expect that additional publishers will commence requiring wwPDB validation reports as part of their manuscript review process.

### User Support

To assist the structural biology and wider scientific community in interpreting the valuable information contained in wwPDB validation reports, the OneDep team has made available an extensive set of documentation materials at https://wwpdb.org/validation/validation-reports. These materials include explanatory notes for each kind of validation report (X-ray, NMR, and 3DEM), frequently asked questions, and instructions for use of the web-service API.

## Discussion

### Outcomes

**Figure 3:**
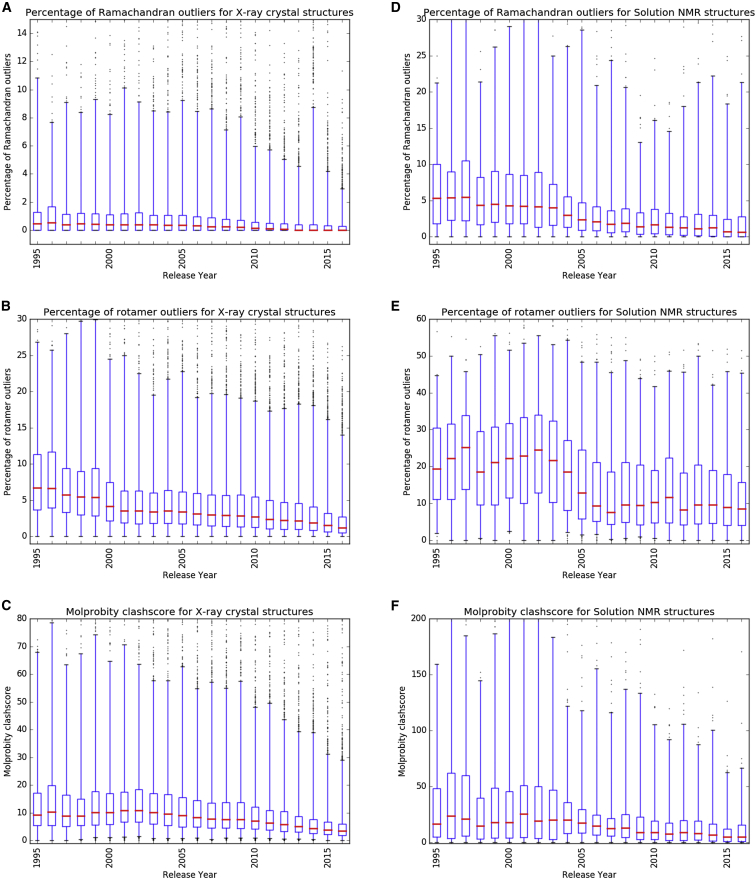
Trends in Geometric Quality Metrics for Protein Structures in the PDB Trends between 1995 and 2016 of geometric validation scores for X-ray crystal and NMR entries in the PDB as reported by MolProbity (Chen et al., 2010). (A–C) Validation metrics for X-ray crystal structures: (A) Ramachandran outliers; (B) rotamer outliers; (C) clashscore. (D–F) Metrics for well-defined regions of Solution NMR structures: (D) Ramachandran outliers; (E) rotamer outliers; (F) clashscore. In each plot, the thick red line represents the median value of each metric for the given year, the box shows the quartile range (25%–75%), and the whiskers show the 1%–99% range. The worst and the best 1% of entries (outside of the whisker range) are plotted as dots.

Introduction of wwPDB validation reports for structures determined by X-ray crystallography, NMR, and 3DEM coincided with growing awareness of the importance of validation in each of the experimental communities. The X-ray crystallography community in particular has developed, over a period of more than 25 years, sophisticated validation tools for analysis of experimental data and atomic models, and of the fit between the two. The NMR community has also made significant advances in the validation arena in recent years. The trends described here reflect a growing maturity of structural biology as a field. Figure 3 documents that geometric quality scores for X-ray crystal structures of proteins have improved over the past decade, as the tools for structure determination evolved and structure validation became more commonplace. It was observed 15 years ago (Kleywegt and Jones, 2002), when data deposition was less common, that the “tendency of macromolecular crystallographers to deposit their experimental data is strongly negatively correlated to the free *R* value of their models.” Thus, another contributing factor to the improving statistics may be the fact that deposition of experimental data has become mandatory since then. This important development enabled better validation of structures, calculation of electron density maps for all crystal structures (e.g., in EDS [Kleywegt et al., 2004]), and recalculation of structural models (e.g., PDB_REDO [Joosten et al., 2014]).

Ramachandran analysis is perhaps the best-known and most widely used geometric quality metric for experimentally determined models. Figure 3A shows that the distribution of the fraction of residues in an entry classified as Ramachandran outliers remained relatively constant until approximately 2005, at which time the distribution started to narrow. Only 25 of the released X-ray crystal structures deposited to the PDB in 2016 (0.2% of entries deposited in that year) had more than 5% Ramachandran outliers. Similar trends are observed for the fraction of residues modeled in non-rotameric conformations and for the clashscore of X-ray crystal structures (Figures 3B and 3C). More detailed statistical analyses of the PDB archive show that X-ray structure quality assessed over 2-year intervals improved between 2012–2013 and 2014–2015 (Shao et al., 2017).

For NMR entries, the analysis of validation metrics reveals fewer trends. There has been no perceptible change in the fraction of Ramachandran outliers, residue side chains modeled in non-rotameric conformations, or clashscores since 2006, and the observed distributions of these metrics are considerably wider than seen for X-ray crystal structures (Figures 3D–3F). Nevertheless, the highest-quality NMR structures compare well with crystal structures on these three metrics.

**Figure 4:**
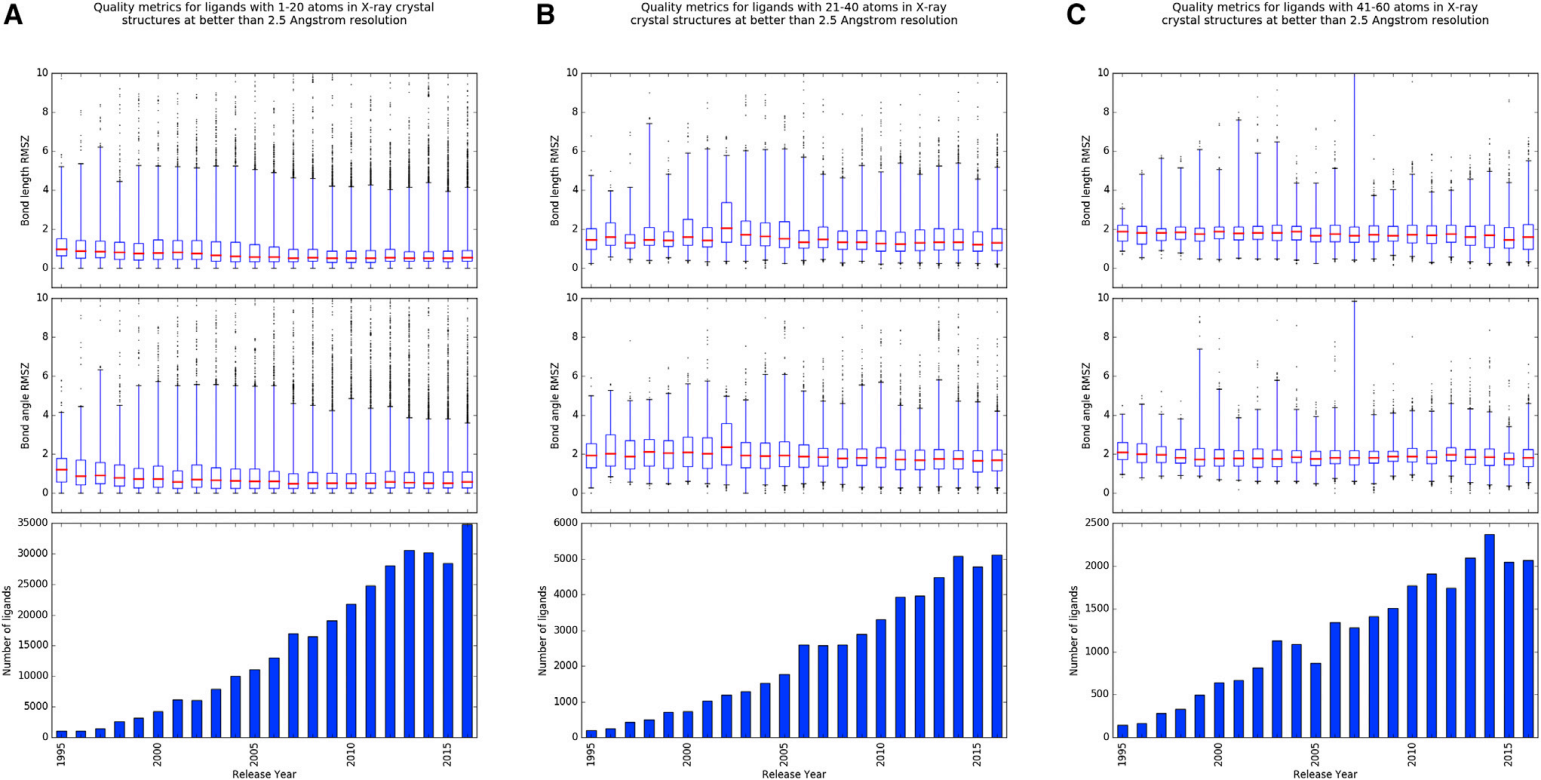
Trends in Geometric Quality Metrics for Small Molecules in the PDB Trends between 1995 and 2016 of bond length and bond-angle RMSZ metrics as determined by Mogul (Bruno et al., 2004) for small molecules in X-ray crystal structures in the PDB at better than 2.5 Å resolution. (A) ligands with 1–20 non-hydrogen atoms; (B) ligands with 21–40 non-hydrogen atoms; (C) ligands with 41–60 non-hydrogen atoms. In each box plot, the thick red line represents the median value per year, the box shows the interquartile range (25%–75%), and the whiskers show the 1%–99% range. Values outside of the whisker range are plotted as dots. In each plot, the top panel shows the bond length RMSZ metric, the middle panel shows the bond-angle RMSZ metric, and the bottom panel shows the number of such ligands deposited in each year.

Figure 4 shows that the quality of bond lengths and bond angles for ligands and small molecules deposited to the PDB, as assessed with Mogul, has remained unchanged during the past decade. The wwPDB, having become keenly aware of this issue, convened the first wwPDB/CCDC/D3R Ligand Validation Workshop in 2015. This workshop brought together co-crystal structure determination experts from academia and industry with X-ray crystallography and computational chemistry software developers with the goal of discussing and developing best practices for validation of co-crystal structures, editorial/refereeing standards for publishing co-crystal structures, and recommendations for ligand representation across the archive. These recommendations have been published (Adams et al., 2016) and were endorsed by the wwPDB X-ray VTF at its most recent meeting in November 2015. Implementation of the recommendations is under way.

### Future Prospects

The OneDep validation module will continue to be developed and improved as the wwPDB partnership receives further recommendations from the expert VTFs for X-ray, NMR, and 3DEM, the OneDep system is refined, and feedback is received from PDB depositors and users alike.

Analyses of wwPDB biocuration efficiency (Young et al., 2017) have suggested that further improvements could be made by encouraging the use of the stand-alone wwPDB validation server. The wwPDB biocurators note that one of the major reasons for depositors to re-refine their models after the first round of biocuration is poor validation metrics pertaining to ligands. This realization informs the ongoing wwPDB efforts to provide richer information about the quality of ligands in the preliminary reports, including an encouragement to submit the refinement dictionaries used by depositors. A recent improvement to the OneDep deposition web pages allows highlighting of major issues pertaining to polymer geometry from the validation reports with the intention of providing this information in a more accessible form. Preliminary data indicate a reduction in the number of data replacements following this change. Table 2 illustrates the bidirectional interaction between depositors and the wwPDB OneDep system. The wwPDB partners strongly encourage depositors to first use the stand-alone validation server and correct their structural model as much as possible prior to deposition. Depositors are also strongly advised to address issues raised by the wwPDB biocuration staff prior to release of the PDB entry.

The wwPDB validation reports for NMR structures do not yet include analysis of NMR restraints. To achieve this goal, the wwPDB in partnership with Leicester University has convened a working group for standardization of restraint representation (Gutmanas et al., 2015). The resulting NMR Exchange Format (NEF) will be supported by all major NMR software packages for structure determination and will be unambiguously convertible to the NMR archival format (NMR-STAR). The NMR-STAR dictionary has been updated to handle the data in NEF format, and dictionary version 3.2 has been released in January 2017. A bidirectional translator to interconvert NEF and NMR-STAR files is now also available. The wwPDB validation pipeline will be extended to include analysis of restraint data and of the fit between atomic model and restraints.

The wwPDB validation reports for 3DEM structures currently include only assessment of geometric parameters for the map-derived atomic coordinates. In the near future, we will add basic information about the experimental map and map-model fit, integrating some of the features from the EM map visual analysis software (Lagerstedt et al., 2013). Recent technological breakthroughs in 3DEM have already led to a rapid increase in the number of depositions of electric potential maps in EMDataBank and atomic models in the PDB. The wwPDB and EMDataBank partners are leading community efforts to define the information to be collected at deposition and to solve challenges of validation of 3DEM maps, models, and the fit between the two (Henderson et al., 2012). At a recent wwPDB PDBx/mmCIF working group meeting, a decision was taken to convene a Subcommittee for Electron Microscopy; we also plan to reconvene the EM VTF to obtain further recommendations.

## Author Contributions

Major contributors to this project are S.G., E.S.G., P.M.S.H., A.G., J.D.W., Z.F., and H.Y. The X-ray validation pipeline was implemented by S.G., Z.F., H.Y., O.S.S., and J.D.W. The NMR validation pipeline was implemented by P.M.S.H., A.G., Z.F., O.S.S., and S.M. The 3DEM validation pipeline was implemented by E.S.G. and O.S.S. The validation pipeline was integrated in the OneDep deposition and biocuration system by J.D.W., T.J.O., E.P., Z.F., E.S.G., P.M.S.H., L.M., O.S.S., and J.M.B. The stand-alone validation web server was implemented by E.S.G., E.P., T.J.O., P.M.S.H., and J.D.W. The validation web-service API was implemented by J.D.W. Annual report recalculations were performed by S.G. and O.S.S. Testing of integrated systems and feedback on the report content were provided by S.S., J.Y.Y., J.M.B., G.S., A.M., C.S., E.P., B.P.H., M.R.S., C.L.L., A.P., A.G., Y.I., N.K., K.B., E.L.U., and R.Y. Project management was provided by A.G., A.P., M.Q., J.D.W., and J.Y.Y. Overall project direction was provided by J.L.M., H.N., H.M.B., S.K.B., S.V., and G.J.K. The manuscript was written by A.G., J.Y.Y., J.D.W., C.L.L., J.M.B., O.S.S., and A.P.
